# Surgical Repair of Nasal Septal Perforation by Mucosal Rotational Flap and Interposition of Cryopreserved Amniotic Membrane

**DOI:** 10.30476/DENTJODS.2021.90431.1489

**Published:** 2022-09

**Authors:** Farrokh Farhadi Shabestari, Reza Khorshidi Khiavi, Mohsen Hashemi, Tannaz Abdollahzadeh Baghaei

**Affiliations:** 1 Dept. of Oral and Maxillofacial Surgery, Faculty of Dentistry, Tabriz University of Medical Sciences, Tabriz, Iran; 2 Dept. of Orthodontics, Faculty of Dentistry, Tabriz University of Medical Sciences, Tabriz, Iran

**Keywords:** Amnion, Nasal septal perforation, Nasal surgical procedures

## Abstract

**Statement of the Problem:**

The surgical repair of nasal septal perforation (NSP) has always been a challenging procedure and no consensus has been made about a definitive protocol.

**Purpose:**

In the current study, we investigated the use of cryopreserved amniotic membrane with mucosal rotational flap for the surgical repair of NSPs.

**Materials and Method:**

In this prospective clinical study, 12 patients with symptomatic NSP underwent primary surgical repair, between December 2018 and October 2019. The surgical procedure comprised of a rotational flap on one side of the defect and cryopreserved amniotic membrane as an interpositional graft in the mucoperichondrial pocket on the other side. The patency of defect was checked at a follow-up appointment at least 3 months after surgery.

**Results:**

Successful repair was perceived in 10 of 12 (83%) of patients. Reperforation occurred in two patients but the size of the defect was smaller than the original one. All of the patients reported elimination of all symptoms associated with NSP.

**Conclusion:**

The use of cryopreserved amniotic membrane as an interpositional graft accompanied by a mucosal rotational flap seems to be efficient in alleviating the symptoms of NSP and closure of the defect.

## Introduction

Nasal septal perforation (NSP) is a relatively common anatomic defect of the nasal septum and affect nearly 1% of the general population [ 1 - 2 ]. The etiology of N-SP is varied, but is often the result of trauma, inhaled irritants, neoplasm and infectious or inflammatory dis-orders [ 3 ]. For symptomatic septal defects, symptoms include epistaxis, discharge, nasal obstruction, crusting, dryness, pain and whistling. However, around 62% are asymptomatic, which do not require closure [ 4 ]. Various techniques for repair of these perforations have been suggested including flaps [ 5 ], tissue expansion [ 6 ], and the use of allogeneic tissues, and biomaterials [ 7 - 8 ]. 

### Human amniotic membrane

Human amniotic membrane (HAM) is growingly being used as an allograft material in surgical procedures related to the genitourinary tract, skin, brain, and head and neck, particularly in ophthalmological reconstructive surgery. Some papers have recently suggested the use of HAM in oral and maxillofacial surgery [ 9 - 10 ]. It has been used for increasing the rate of survival of ischemic random pattern flaps [ 11 ], vestibuloplasty [ 12 - 13 ], implant surgery [ 14 ], closure of oronasal fistula [ 15 ], and replacement of diseased nasal mucosa [ 16 ], arthroplasty [ 17 ], and gingival wound healing [ 18 ]. Moreover, HAM is easily available and reasonably priced regarding its harvesting and processing to a medical product (for instance, testing for viral infections of the donors and cryopreservation) [ 19 ]. In the current study, we investigated the use of multilayered HAM for the surgical repair of NSPs.

## Materials and Method

**Figure 1:**
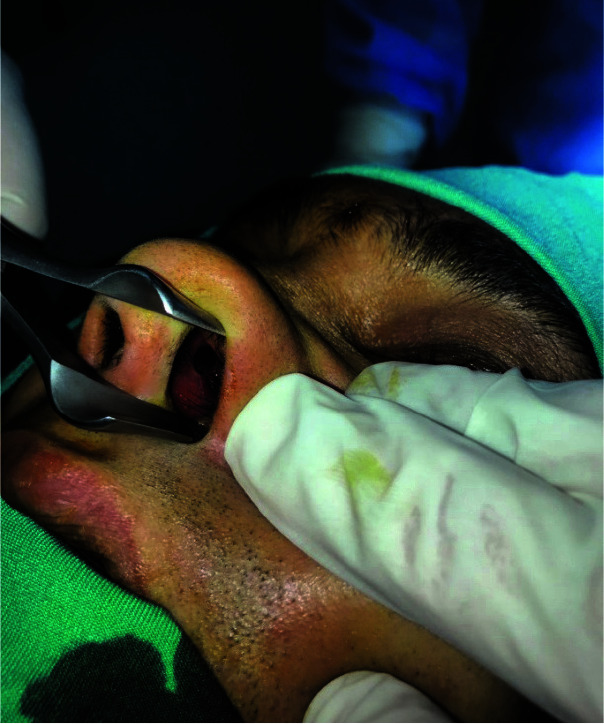
Diameter of nasal septal perforation was measured preoperatively

After acceptance by the Ethics Committee of Tabriz University of Medical Sciences (code: IR.TBZMED. REC.1397.662), this study was conducted on 12 symptomatic patients with NSP (8 male and 4 female subjects). Patients with any form of tobacco use, defects larger than 2 cm, and an underlying systemic disease such as autoimmune problems were excluded. None of the patients had unsuccessful previous surgical repair. The participants underwent surgical closure of the perforation between December 2018 and October 2019, at Oral and Maxillofacial Department of Tabriz University of Medical Sciences. Written informed consent was obtained from both the participants and the mothers donating HAM. The situation of all perforations was at the anterior part of septum. The size of perforation was determined by the largest diameter of the defect and was measured by a ruler in a nasal examination ( Figure 1). During the presurgical period, daily irrigation of the nasal cavity was performed by sterile saline for 2 weeks. Nasal mupirocin ointment (Mupirocin 2%, Sina Daru Co., Tehran, Iran) was applied after irrigation.

### Surgical technique

**Figure 2:**
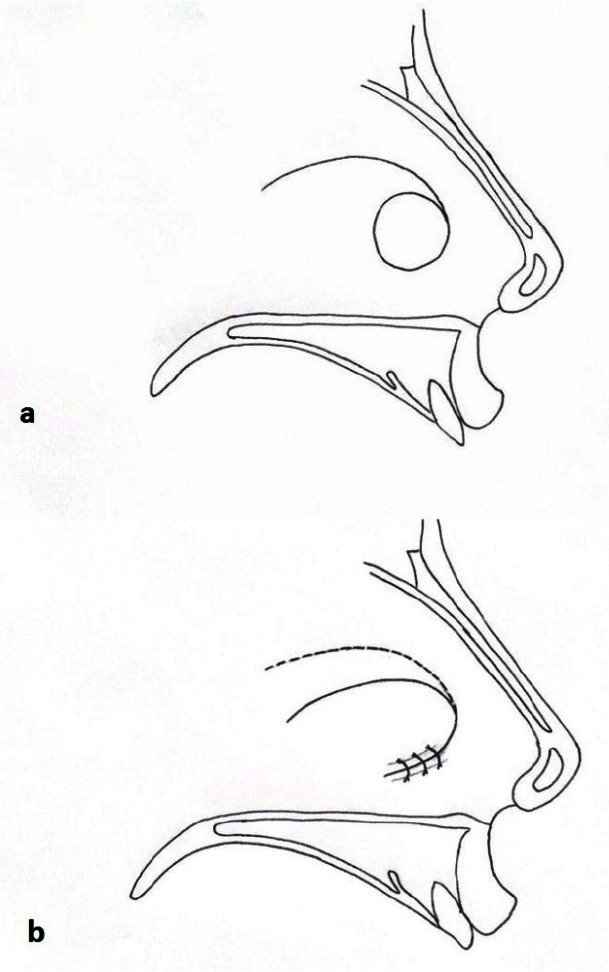
**a:** Diameter of nasal septal perforation was measured preoperatively. **b:** Rotational flap based on posterior nasal artery

The surgery was conducted by closed approach. Mucososal rotational flap was planned to be raised on the side with a more patent nostril for better access and the mucoperichondrial flap on the contralateral side. At the beginning, 1% lidocaine with 1: 100,000 epinephrine was injected in the septal area, and then oxymetazoline-soaked cotton was placed intranasally. The periphery of the defect was sharply incised. The incision for the mucosal rotational flap starts in the posteroinferior portion of the septum, continues in an anterosuperior direction, and reaches to the anterior border of the perforation. The blood supply of the flap is provided by a branch of the sphenopalatine artery (the posterior nasal artery). After elevation of the rotational flap, it was sutured to the periphery of the defect with chromic gut ( Figure 2).

**Figure 3:**
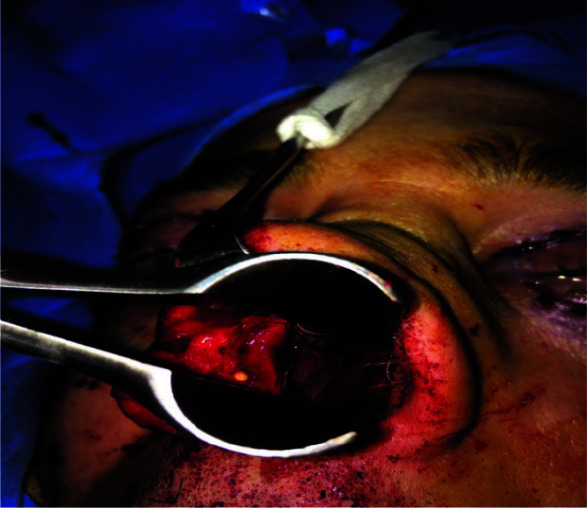
Rotational mucosal flap was performed on one side of septum

**Figure 4:**
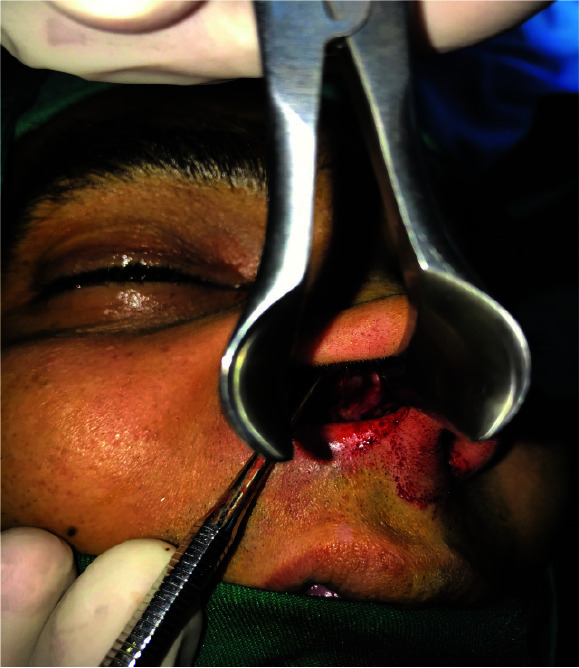
Preserved amniotic membrane was placed in the mucoperichondrial pocket on the other side of septum

The cartilage was left exposed on the posterior superior aspect of the flap pedicle (Figure 1). On the contralateral side of the septum, a hemitransfixion incision was made and a mucoperichondrial flap was elevated. Then, cryopreserved HAM was cut to fit the perforation size and was placed in the mucoperichondrial pocket ( Figure 3). Finally, the hemitransfixion incision was sutured and internal splints coated in tetracycline 3% ointment, were placed intranasally ( Figure 4). The internal splints were removed two weeks later.

### Follow-up

All patients were followed at least 3 months after surgery to check the closure of the defect artery.

## Results

The patients were aged between 18 and 40 years (mean: 32.5). Mean defect size was 1.49 cm (range: 1 to 2cm). Epistaxis and dryness were the most common symptoms. Successful closure of the defect was observed in 10 out of 12 patients, which means a success rate of 83%. Table 1 depicts the data of all cases and results of surgical repair of each case. The closure rate is 100% in cases with a defect smaller than 1cm and 80% in cases with a defect size of 1-2cm. A considerable reduction in the size of defect was noted in the two cases with reperforation. Total healing of normal mucosa and elimination of crusting was detected in all of the cases whose defect was completely closed. All of the cases, including the two cases with reperforation, reported elimination of all symptoms associated with NSP during the post-surgical period.

**Table 1:** Patient information, and the results of surgical repair

Patient No.	Sex	Age	Cause of perforation	Size	Result	Follow-up duration (months)
1	M	27	Unknown	1.5 cm	Closure	12
2	M	30	Trauma	1.3 cm	Closure	11
3	F	35	Septo/septorhinoplasty	2 cm	Closure	11
4	M	32	Trauma	2 cm	Closure	10
5	F	34	Septo/septorhinoplasty	1.4 cm	Closure	10
6	M	38	Septo/septorhinoplasty	1.6 cm	0.8 cm Defect	10
7	M	33	Trauma	1 cm	Closure	8
8	M	40	Trauma	2 cm	0.6 cm Defect	7
9	F	26	Septo/septorhinoplasty	1.2 cm	Closure	7
10	F	29	Septo/septorhinoplasty	1 cm	Closure	5
11	M	37	Septo/septorhinoplasty	1.5 cm	Closure	4
12	M	29	Trauma	1.4 cm	Closure	3

## Discussion

NSP occurs in cases with blood supply deprivation due to rupture of the mucoperichondrium on both sides of the septal cartilage. This condition is possibly encountered in patients with history of trauma, surgical manipulation of the nasal septum, various drugs, and tumors [ 20 ]. In this study, NSP was mainly associated with surgical manipulation and trauma. Prevention is the best way for management of NSP [ 21 ].

NSP typically does not cause any symptoms, especially the ones located posteriorly. These defects tend to be smaller and less symptomatic due to better humidification in this area. On the other hand, the perforations located anteriorly are presumably associated with symptoms such as a sense of dryness, as it was in all of our cases. Fortunately, manipulation of mucoperichondrium and managing flaps is much easier in anteriorly posioned defects. Epistaxis and dryness were the most common complaints in this study.

The main purpose of treatment should be improvement of the quality of life by alleviating the symptoms. 

The first line of treatment is aggressive irrigation of the nasal cavity and topical moisturizing ointments [ 7 ]. The use of septal buttons may cause even more widening of the defect and therefore could worsen the symptoms [ 22 ]. Surgical intervention is the final line in the treatment of NSP, provided when the cause of the defect is not a persistent source such as systemic condition like autoimmune disorders or cocaine abuse [ 23 ]. Many surgical approaches have been reported, none of which has been advocated by high-level evidence as the gold standard [ 20 ]. This is mostly due to the variety of applied approaches and insufficient sample size. The reported follow-up duration is also important. Some defects may seem to be successfully closed but reperforation may occur after a while [ 24 ]. 

A rotational flap design was considered to close the defect in this study. Rotational flaps as compared to bipedicled flaps are more easily advanced without adding tension to the flap [ 25 ]. There is no controlled study to compare flap-only closure with flaps plus interpositional grafts, but higher closure rates are reported by studies utilizing local mucosal flaps and interposition grafts compared to studies using only local mucosal flaps to close perforations [ 25 - 30 ]. There are numerous alternatives for the graft material. Autografts such as temporal fascia, periosteum, bone of nasal septum, and allografts such as human dermis has been used [ 31 ]. Autografts like temporal fascia [ 26 , 32 ] and mastoid periosteum [ 29 , 33 ] have shown high success rates but donor site morbidity remains as a drawback. Septal graft also doubles this problem by reducing the support of the septal area and therefore increasing the risk of reperforation or even formation of a new defect [ 34 ]. Acellular human dermal graft has also been suggested as it is available in sufficient amounts, eliminates morbidity, and is thicker than grafts such as temporal fascia. Studies have reported miscellaneous results about the advantage of this graft material [ 7 , 35 - 36 ]. Human amniotic membrane (HAM) is a newly introduced graft material, which is routinely used in ophthalmology. It has recently been used in oral and maxillofacial surgery for healing of the soft tissue around dental implants, healing of intraoral wounds, vestibuloplasty, and treatment of gingival recession [ 10 , 37 - 38 ]. Properties such as improvement of intraoral and extraoral epithelialization, pain reduction, and scarring make HAM a suitable graft material in defects such as NSPs [ 10 ]. 

In our study, 83.3% of all cases showed complete closure at the follow-up appointment. The closure rate was 100% in cases with a defect equal to or smaller than 1 cm and 80% in cases with a larger defect. This is a higher success rate compared to Delaney and Kridle [ 39 ] who reported a success rate of 90% for perforations smaller than 1cm and 73% for larger perforations. Reperforation occurred in two cases. Despite the reperforation of these cases, all of the cases reported no symptoms in the follow-up appointment, which means that the main goal of alleviation of symptoms was achieved. This results show a promising effect for application of HAM as an interpositional graft in surgical repair of NSPs. Both of the cases with reperforation had relatively large defects (more than 1.5cm). One of these cases had a history of nasal spray consumption, which may have limited the blood supply of the adjacent mucosa irreversibly and thereby have worsened the healing potential of local soft tissue. The follow-up duration was not the same for all of the cases. A longer follow-up period would be beneficial in the future studies as reperforation is possible after a while. Unfortunately, due to the limited number of cases, any statistical evaluation of the results was not possible, but it seems that more experience with HAM as graft material in NSP repair would possibly show high success rates and complete elimination of the symptoms. Further studies with this graft material would be advantageous.

## Conclusion

Surgical repair of NSPs is a challenging procedure. Considering the stimulation of epithelialization by HAM as a biomembrane, it seems that using it as a graft material accompanied by a mucosal rotational flap would be beneficial. Although the rate of complete defect closure was not 100%, elimination of the symptoms in all cases indicates that this technique may help us for a better management of the patients suffering from NSP.

## Conflict of Interest

 The authors declare no competing interests with regards to the authorship and/or publication of this article. 
